# Development of a Rat Model of Sick Sinus Syndrome Using Pinpoint Press Permeation

**DOI:** 10.1155/2018/7487324

**Published:** 2018-11-18

**Authors:** Hong-bin Zhong, Ting-jun Wang, Gui-li Lian, Chang-sheng Xu, Hua-jun Wang, Liang-di Xie

**Affiliations:** ^1^Fujian Hypertension Research Institute, The First Affiliated Hospital of Fujian Medical University, Fuzhou, China; ^2^Xiang'an Branch, The First Affiliated Hospital of Xiamen University, Xiamen, Fujian, China

## Abstract

**Objective:**

Sick sinus syndrome (SSS) is one of the most common causes of cardiac impairment necessitating pacemaker implantation. However, studies of SSS pathogenesis are neither comprehensive nor conclusive due to limited success in achieving a stable rat SSS model. Here, we modified pinpoint press permeation to establish a stable rat SSS model.

**Methods:**

We randomly assigned 138 male Sprague-Dawley rats into three groups: normal control (n = 8), sham (n = 10), and SSS (n = 120). Postoperatively, the SSS group was further divided into SSSA (n = 40), SSSB (n = 40), and SSSC (n = 40), based on reduction in heart rates by 20–30%, 31–40%, and 41–50%, respectively. We also assessed histomorphological characteristics and hyperpolarization-activated cyclic nucleotide-gated cation channel 4 (HCN4) expression in the sinoatrial node (SAN) at 1, 2, 3, and 4 weeks after surgery.

**Results:**

Mortality was statistically higher in SSSC compared to SSSA and SSSB (7.5%* versus* 90.0% and 87.5%; P < 0.05). Heart rate in SSSA was gradually restored to preoperative levels by week 4 after surgery. In contrast, heart rate in SSSB was stable at 2–3 weeks after surgery. However, we observed that the tissues and cells in SAN were severely injured and also found a time-dependent increase in collagen content and atrium myocardium in SSSB. HCN4 expression was significantly reduced at all 4 time points in SSSB, with statistically significant differences among the groups (P < 0.01).

**Conclusion:**

We successfully developed a rat SSS model that was sustainable for up to 4 weeks.

## 1. Introduction

Sick sinus syndrome (SSS) is one of the most common causes of sudden cardiac death, characterized by refractory bradyarrhythmia, and necessitates implantation of a permanent pacemaker [1–3]. According to histological and physiological studies, abnormal cardiac impulse formation or conduction disturbance is believed to be the main pathological mechanism leading to SSS [4]. However, the precise pathogenesis of SSS remains poorly understood since there has been little success in establishing a stable animal model of the disease. To this end, our study aimed to identify a method to develop a stable rat SSS model.

Several procedures to develop an SSS animal model have been reported, primarily involving physical or chemical impairment of the sinoatrial node (SAN). Physical methods of impairing the SAN include cryocoagulation of the sinus node area [5], radio frequency ablation [6], and right coronary artery ligation [7]. However, physical methods have significant limitations, including procedural intricacy, unwarranted complications, low success rates, and unsuitability for smaller animals. Chemical damage using formaldehyde or sodium hydroxide wet compression has attracted wider use with high success rates and fewer complications compared to physical methods and therefore is commonly used in laboratories to establish animal disease models [8–10].

Numerous attempts to establish experimental animal SSS models have been recently reported [5–10]. However, a majority of these studies were performed with larger animals, such as pigs, rabbits, or canines. Rats have been largely neglected as a viable model of SSS since the SAN in rats is concealed and is difficult to view with the naked eye. Thus, limited success has been reported for establishing rat SSS models. There is a pressing need to develop and validate rat SSS models for use in pathogenetic studies, which is the goal of the current study.

Here, we describe a method of pinpoint press permeation to develop a rat SSS model and then evaluate its stability for investigating SSS pathogenesis. We also compare the survival rates, heart rate changes, histomorphological manifestation, and hyperpolarization-activated cyclic nucleotide-gated cation channel 4 (HCN4) protein expression levels in SSS, sham, and normal control rats. Subsequent to chemically induced impairment of the SAN region in our experimental animals, biological samples were collected at different time points to determine the feasibility of the established rat SSS model.

## 2. Materials and Methods

### 2.1. Animals

A total of 138 Sprague-Dawley rats (12-week-old males, weighing 250 ± 10 g) were purchased from the Shanghai SLACCAS Laboratory Animal Co. (Shanghai, China; Certificate No. 20070005). Five rats were housed per cage and all rats had free access to tap water and food. Rats were housed at 22 ± 2°C, 55 ± 5% humidity, and in a 12-hour artificial light/dark cycle. All animal experiments were approved by the Animal Ethics Committee of Fujian Medical University of China.

### 2.2. Drugs and Instruments

Materials and kits were procured as follows: 2% pentobarbital sodium and 20% sodium hydroxide solution (YoubangChe Co., Zhejiang, China); 10% neutral buffered formalin, Harris hematoxylin dye, eosin dye, and Ponceau Fuchsin acid liquid (SBJBio Co., Nanjing, China); Masson staining kit and immunohistochemistry kit (Shuobo Biotechnology, Shanghai, China); rabbit anti-HCN4 antibody (ab66501; Abcam, USA); goat anti-mouse IgG and goat anti-rabbit IgG (Beijing Zhongshan Golden Bridge Biotechnology Co., Beijing, China); SMZ445 microscope and Nikon80i microscope (Nikon, Japan); TKR-200 small animal ventilator (BME Co., Jiangxi, China); and Powerlab multichannel physiological recorder (Ed, Australia).

### 2.3. Establishing an Animal Model

Rats were randomly assigned to three groups as follows: control group (n = 8), sham group (n = 10), and SSS group (n = 120). The SSS group was further divided based on reductions in heart rates: the SSSA group had a 20–30% reduced heart rate (n = 40), the SSSB group had a 31–40% reduced heart rate (n = 40), and the SSSC group had a 41–50% reduced heart rate (n = 40). Eight rats were sacrificed at four time points for further analysis. The time points included the last day of weeks 1, 2, 3, and 4 after induction of SAM impairment, represented by SSS1, SSS2, SSS3, and SSS4, respectively.

**Figure 1:**
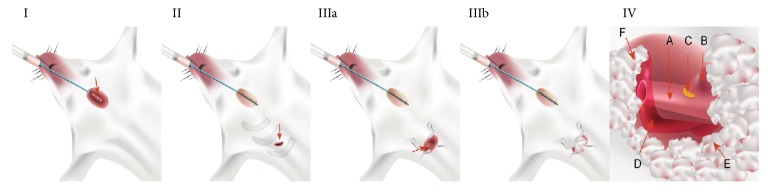
**Establishing a rat SSS model. (I) Endotracheal intubation.** Intubation is evidenced by the use of a pediatric indwelling needle (red arrow), and wound closure with thread suture is shown (red arrow).** (II) Incision.** A diagonal incision along the second intercostal gap in the right edge of the sternum (red arrow) and a blunt separation along the second intercostal gap into the chest are shown.** (III) Exposure of thoracic cave. **A gap was made between the second and the third rib using a blepharostat, exposing the right lung tissue (**IIIa**) on which to place a sterile cotton ball (**IIIb**).** (IV) Exposure of the SAN region.** The vascular coat was cut open and the SAN region was exposed at the junction between superior vena cava and right atrium. Sterile cotton balls were used to cover the right lung and the thymus. A: superior vena cava; B: right auricle; C: SAN region; D: vascular coat; E: right lung under the sterile cotton balls; F: thymus under the sterile cotton balls.

All procedures were performed under a surgical microscope by the same investigator. After recording electrophysiological indexes, rats were anesthetized with an intraperitoneal injection of 2% pentobarbital sodium (50 mg/kg body weight) for 2-3 min and placed on an operation table. Skin preparation and disinfection of the chest were performed. Surface electrocardiogram was continuously monitored using a BL-420F data acquisition and analysis system. To confirm establishment of the rat SSS model, a custom-made device was used as previously described [11]. A provision of respiratory support to experimental animals was ensured using tracheostomy with a small ventilator (Figure 1 I). Surgical procedures involved the following steps: (1)* Incision*: 1.0 cm diagonal incision was made at the second intercostal gap along the right edge of the sternum, and intercostal muscles were bluntly dissected along the second intercostal gap up to the chest cave using tweezers, ensuring no involvement of the rib (Figure 1 II). (2)* Exposing the SAN region*: the chest wall was opened using an eyelid retractor (Figure 1 IIIa). Because the SAN region lies beneath the right lung and thymus, aseptic cotton balls were used to isolate the surrounding tissues, including the right lung, the thymus, and the right auricle (Figure 1 IIIb). (3)* Impairing the SAN region*: in the SSS group, a 2-mm cotton ball and 1 mL 20% sodium hydroxide in a custom-made syringe were used to perform pinpoint press permeation in the SAN region for 3–5 min (Figure 1 IV). In the sham group, a 2-mm cotton ball and normal saline were used. In the control group, press permeation was performed without any solution. Establishment of the rat SSS model was confirmed based either on the reduction of heart rates by 20–30% (SSSA group), 31–40% (SSSB group), and 41–50% (SSSC group) or by the appearance of sinus arrest and nodal escape.

### 2.4. Hematoxylin and Eosin (HE) and Masson Staining

**Figure 2:**
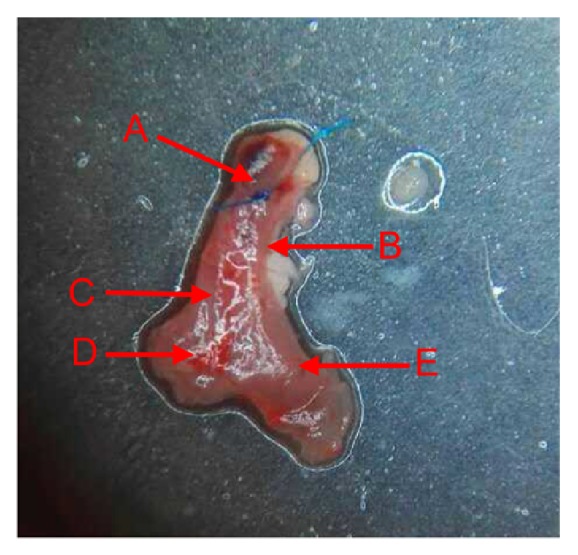
**SAN region. A: **a knot made using a 7-0 thread;** B:** superior vena cava;** C: **crista terminalis;** D: **inferior vena cava;** E:** right auricle.

Subsequent to confirming impairment of the SAN region, the heart was exposed (Figure 2) and a 3–5 mm knot was made using a 7-0 thread from the telecentric end of the superior vena cava to orient the tissue for sectioning. The heart was removed immediately, rinsed with saline, and sectioned. Transverse serial sections (4-*μ*m-thick) of the heart were fixed with 4% paraformaldehyde for up to 48 h, dehydrated using increasing concentrations of ethanol, air-dried, and embedded in paraffin. Two adjacent sections were removed for HE and Masson staining and observed using light and inverted fluorescence microscopy.

### 2.5. Immunohistochemical Study

As previously described [11, 12], heart tissue samples were embedded in paraffin and sectioned (4 *μ*m) before immunohistochemical analysis. After dewaxing and debenzolization, rabbit anti-HCN4 antibody (ab66501; Abcam, USA) was added to the sections at a dilution of 1:200 at 4°C overnight, followed by washing with tris buffered saline (TBS) at room temperature, with subsequent addition of anti-rabbit horseradish peroxidase (Beijing Zhongshan Golden Bridge Biotechnology Co.) at 37°C for 2 h. Finally, the sections were counterstained with hematoxylin, dehydrated, and mounted using neutral gum.

### 2.6. Statistical Analysis

Data are expressed as mean ± standard deviation. One-way ANOVA, Chi-square test, and least significant difference test were used for comparisons of means among groups, categorical variable data, and pairwise means within a group, respectively.* P *values < 0.05 represent statistically significant differences. We used SPSS 18.0 software for automated data analysis.

## 3. Results

### 3.1. Relationship between Reduced Heart Rate and Survival Rate

**Figure 3:**
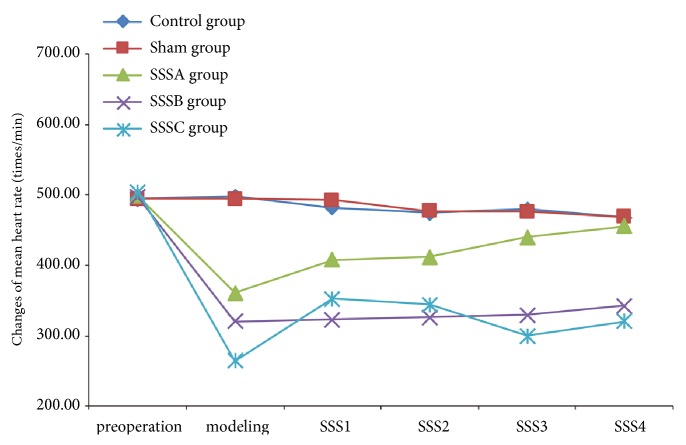
**Changes in heart rates at different time points. **Compared to the control group, heart rates in the SSSA group were gradually restored to preoperative values within 4 weeks. For the SSSB group, heart rates first decreased by 31–40% and stabilized after 2-3 weeks but were lower than the preoperative values. Heart rates of surviving rats in the SSSC group were restored by 35% of the preoperative values within 4 weeks.

Survival rates within 4 weeks of the procedure were ~90% in the SSSA group, with heart rates returning to normal levels by week 3. While survival rates in the SSSC group within 1 week of the procedure were only below 7.5%, heart rates were restored to 65–70% of normal values during week 3. Survival rates within 4 weeks of the procedure were ~87.5% in the SSSB group, with heart rates returning to stable levels (Table 1; Figure 3).

### 3.2. Histomorphological Characteristics of the SAN in the SSSB Group

**Figure 4:**
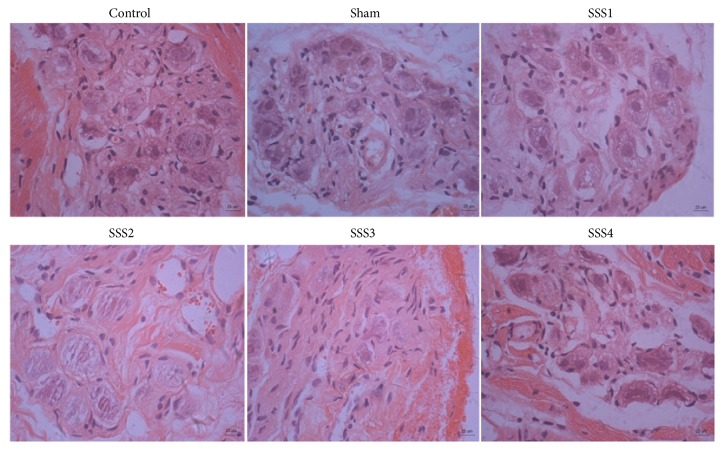
Changes in morphology and structure of SAN cells at different time points (HE staining, scale bar 25 *μ*m).

In SSSB rats with stable heart rates, histomorphological evaluation indicated a clear ganglion cell structure with distinct boundary lines, and we also observed a distribution of epithelial-like cells in the ganglion cell spaces. In the SSS1 group, the structure of ganglion cells was clear, with small amounts of proliferating connective tissue and epithelial-like cells appearing with enlarged cell nuclei in the ganglion cell spaces. However, in the SSS2, SSS3, and SSS4 groups, the ganglion cells were undefined with vague boundaries and large amounts of connective tissue proliferation. In addition, we observed vacuolation in the SSS3 and SSS4 groups (Figure 4).

### 3.3. Increased Atrial Fibrosis in the SSSB Group

Our findings are similar to those reported previously [11]. In SSSB rats with stable heart rates, there was a time-dependent increase in collagen as determined by Masson staining. Collagen in the myocardial tissue of rats in the SSS1 group was higher compared to the sham group (P < 0.01), while the presence of collagen in the SSS2, SSS3, and SSS4 groups was significantly higher (P < 0.01).

Similarly, we observed derangement of the right atrial cardiomyocytes in rats sacrificed at week 1 after the procedure (SSS1). This was accompanied by a small number of hyperplastic cells and increased myocardial tissues in the interstitial myocardium. Hyperplastic tissues in the interstitial myocardium were further increased, as indicated by green staining, and myocardial tissues were further increased by week 2 (SSS2). Large patches of myocardial tissues were seen at weeks 3 and 4 (SSS3, SSS4). Collagen levels increased in a time-dependent manner in the atrial tissues of the SSSB group compared to the sham group. Thus, collagen levels in SSS rats were significantly increased at 1 week (P < 0.01) and further increased at 3 and 4 weeks (P < 0.01) after surgery.

### 3.4. Changes in HCN4 Expression in the SSSB Group

**Figure 5:**
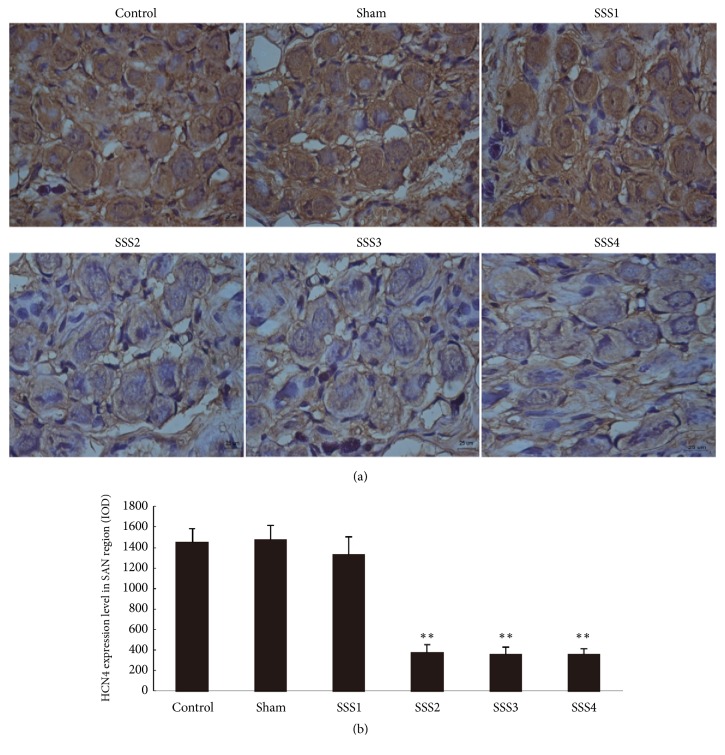
Comparison of HCN4 expression levels after immunohistochemical staining (a) immunohistochemistry; scale bar 25 *μ*m; comparison based on integral optical densities (b). *∗∗P *< 0.01 versus control and sham groups.

Using immunochemical analysis, we found that HCN4 protein expression in the SAN cells was regularly arranged with obvious intercellular boundaries. For both control and sham groups, nuclear size was uniform, and flakes or strips of brown/yellow precipitates were seen in the intercellular space. In the SSS1 group, a relative regularity in the arrangement of SAN cells was noted, and intercellular boundaries were still obvious, while some small flakes or strips of brown/yellow precipitates were seen in the intercellular space. In the SSS2, SSS3, and SSS4 groups, only small amounts of filamentous or small strips of brown/yellow precipitates in the intercellular space were detected (Figure 5(a)). However, based on the integral optical densities of HCN4 protein expression, no significant differences were found in the control, sham, and SSS1 groups (P > 0.05). Compared to the sham and control groups, HCN4 protein expression was significantly decreased in the SSS2, SSS3, and SSS4 groups (P < 0.01) (Figure 5(b)).

## 4. Discussion

Development of a stable rat SSS model has remained elusive in cardiovascular research. Here, we describe our method of successfully inducing SSS by means of SAN damage in rats. Importantly, we also demonstrate that our model is sustainable and stable. Specifically, our data indicate that a 31–40% reduction in heart rate following SAN damage qualifies a stable rat SSS model.

Press permeation using both formaldehyde and sodium hydroxide has been commonly used to establish chronic sinus node damage in animal models. In a previous study, a rabbit SSS model was established utilizing 2-mm cotton balls dipped in 30% formaldehyde for fixed-point infiltration of SAN regions, passing from the right margin of the mesosternum through the mediastinum into the thoracic cavity. In this method, a rib bone unneeded for respiratory support was removed, avoiding injury to the pleura. A success rate to 86.7%, which was significantly higher compared to traditional modeling studies, has been reported [8]. However, this method is unsuitable for establishing a rat SSS model, since the SAN in rats is too small to locate with the naked eye. Previous studies have shown that injury to the SAN with formaldehyde can recover within weeks, whereas recovery after injury with sodium hydroxide took longer. Thus, sodium hydroxide is preferred for inducing chronic SAN damage [10]. In the present study, we established a stable rat model that could be utilized for pathogenetic studies of SSS. Using ventilator support, 20% sodium hydroxide was permeated into SAN tissues for 3–5 min. Heart rate changes, histomorphological characteristics, and changes in HCN4 protein expression in the SAN region were observed over four weeks after the procedure. Our experimental data confirm the stability of our rat SSS model.

SSS is a SAN dysfunction characterized by various forms of bradyarrhythmia. The extent of reduced heart rate to establish a functional animal SSS model remains unclear. In a previously reported SSS model, a 30–50% decrease in heart rate, sinus arrest, sinoatrial blockage, or nodal escape was significant outcomes [8, 9]. However, the number, size, location, and morphology of SAN cells vary between animals, indicating that SAN function and tolerance to low heart rate might vary between animal models. Most preliminary experiments suggested that a reduction in heart rate > 40% led to poor survival rates in rats, whereas an initial heart rate reduction < 30% improved chances of survival in rats. In our study, heart rates were reduced by 20–30% (SSSA group), 31–40% (SSSB group), and 41–50% (SSSC group). The survival rate of rats in the SSSB group was approximately 87.5%, with heart rate stability maintained for up to 4 weeks. HCN4, a subtype of the HCN channel protein family, has been identified as the main gene regulating pacemaker function in the SAN [13–15]. Studies have confirmed a relationship between decline in SAN function and reduced HCN4 expression [16, 17]. Therefore, HCN4 expression in the SAN area of rats in the SSSB group was measured to confirm changes in SAN function. Our data indicate that a rat model with a 31–40% reduced heart rate is suitable for establishing a stable SSS model.

The causes of death in our experimental animals included hemorrhage, pneumothorax, and sudden death. Hemorrhage primarily resulted from right atrial appendage, tissue rupture in the superior vena cava or SAN region, or vascular injury after rib removal. Pneumothorax caused by lung tissue rupture was attributed to the use of formaldehyde or sodium hydroxide. Sudden death was likely the result of the sudden decline in heart rate or other unknown reasons. A significantly higher success rate of the animal model in our study could be attributed to one of several reasons: First, incision size was small (1 cm) and ribs were spared despite blunt dissection of the second right intercostal muscles from the thoracic cavity, avoiding damage to the blood vessels and nerves. Second, the SAN region was fully exposed, while we isolated the right lung, thymus, and right atrial appendage with sterile cotton, avoiding injury to the lung tissue and the right atrial appendage and preventing pneumothorax and hemorrhage. Third, the use of a self-devised trocar ring with cotton half-in and half-out avoided damage to any tissue around the SAN region. In addition, tissue dissection under a microscope increased the accuracy of our work.

**Figure 6:**
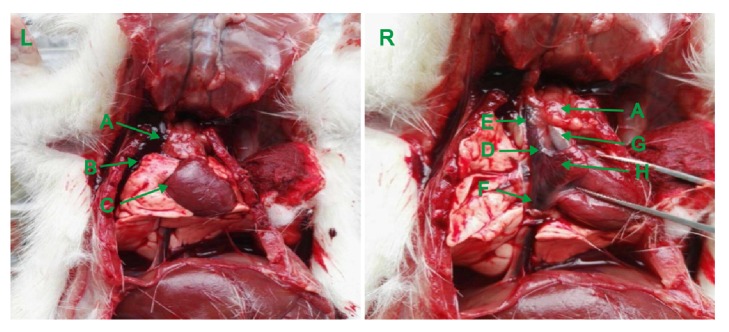
**Anatomy of the SAN region. **(L) SAN covered with thymus and right superior segmental lung. (R) SAN exposed after isolating the right lung, thymus, and right atrial appendage. A: thymus; B: right lung; C: right ventricular; D: SAN region; E: superior vena cava; F: inferior vena cava; G: aorta; H: right atrial appendage.

In a previous report describing a less invasive method, a SSS rat model was established using a custom-made mapping catheter that carried a small cotton ball dipped in 20% sodium hydroxide sans respiratory support. The cotton ball was inserted close to the right edge of the second intercostal sternum, and the location was ensured by observing changes with an electrocardiogram in vitro, eliminating the need to remove a rib or open the chest, yielding a success rate of 70% [9]. Anatomically, the SAN region in a rat is covered by thymus tissue and the right atrial appendage, and the thymus tissue is further wrapped in a layer of epicardium, while the medial part of the right lung is located above the thymus tissue, which is close to the lateral edge in particular (Figure 6, left). Thus, the method described above, we argue, carries a higher risk of in-procedure damage to the lung tissue, potentially leading to pneumothorax. Due to the anatomical restrictions described above, a custom-made syringe carrying a cotton ball cannot reach the SAN area [9] without causing certain damage to the lung and thymus tissue. In addition, the thin epicardium wrapping of the SAN area can only be separated by direct observation. Therefore, we argue that our proposed surgical method to establish the rat SSS model with respiratory support and microscopic assistance is both scientific and feasible.

An ideal SSS animal model should be confirmed by histopathological changes and indication of a variety of arrhythmias. The histopathological changes observed in our rat SSS model mimicked those occurring during clinical presentation of the disease, but various arrhythmias were not observed due to a short observation duration. One limitation of our study was that SAN function and changes in SAN microstructure were not considered. However, histopathological changes and changes in HCN4 expression suggested a malady of SAN function in our animal model.

## 5. Conclusions

Using press permeation to infiltrate 20% sodium hydroxide into the SAN region of rats, we established a stable rat SSS model with a preferred reduction in heart rate of 31–40%. The proposed surgical model that ensures respiratory support to experimental animals and direct observation of tissue samples with microscopy confers several advantages, including higher success rates and reproducibility.

## Figures and Tables

**Table 1:** Comparison of survival rates of modeled rats.

	No. of deaths	No. survived	Survival rate
Control group (n=8)	0	8	100
Sham group (n=10)	0	10	100
SSSA group (n=40)	4	36	90
SSSB group (n=40)	5	35	87.5
SSSC group (n=40)	37	3	7.5*∗∗*

*∗∗P* value < 0.01 versus SSSB group.

## Data Availability

Data that support the findings of this study are included within the article. Requests for access to additional (raw) data will be considered by the corresponding author.
